# Higher Gene Expression Related to Wound Healing by Fibroblasts on Silk Fibroin Biomaterial than on Collagen

**DOI:** 10.3390/molecules25081939

**Published:** 2020-04-22

**Authors:** Tomoko Hashimoto, Katsura Kojima, Yasushi Tamada

**Affiliations:** 1 Silk Materials Research Unit, National Institute of Agrobiological Sciences, 1-2 Owashi, Tsukuba, Ibaraki 305-8634, Japan; 2 Faculty of Human Life and Environment, Nara Women’s University, Kitauoya-nishimachi, Nara 6308506, Japan; 3 Silk Materials Research Unit, Institute of Agrobiological Sciences, National Agriculture and Food Research Organization, 1-2 Owashi, Tsukuba, Ibaraki 305-8634, Japan; 4 Faculty of Textile Science and Technology, Shinshu University, 3-15-1, Tokida, Ueda, Nagano 3868567, Japan

**Keywords:** dermis, silk fibroin, tissue engineering, wound healing

## Abstract

Silk fibroin (SF), which offers the benefits of biosafety, biocompatibility, and mechanical strength, has potential for use as a good biomedical material, especially in the tissue engineering field. This study investigated the use of SF biomaterials as a wound dressing compared to commercially available collagen materials. After human fibroblasts (WI-38) were cultured on both films and sponges, their cell motilities and gene expressions related to wound repair and tissue reconstruction were evaluated. Compared to the collagen film (Col film), the SF film induced higher cell motility; higher expressions of genes were observed on the SF film. Extracellular matrix production-related genes were up-regulated in WI-38 fibroblasts cultured on the SF sponges. These results suggest that SF-based biomaterials can accelerate wound healing and tissue reconstruction. They can be useful biomaterials for functional wound dressings.

## 1. Introduction

Silk fibroin (SF), a natural fiber, has long been used in the medical field as a surgical suture material. Recently, because of SF-based biomaterials’ good biocompatibility, strength, and processability, many have been developed for tissue engineering applications [1]. Applications of SF-based biomaterials for drug delivery devices, artificial vessels, wound dressings, and scaffolds have been anticipated [2,3,4,5]. Regenerated SF can be fabricated from an SF-aqueous solution into various forms such as films, hydrogels, sponges, fibers, nanofibers, and resins such as epoxy composites [6,7,8,9,10]. Our earlier report described the development of an original method for SF sponge preparation using a simple freeze–thawing process, with addition to an SF-aqueous solution of a small amount of water-miscible organic solvents [9]. Our SF sponges have uniform pores and show high water contents because such surfaces are suitable for cell growth and handling, suggesting their usefulness as tissue engineering scaffolds [9]. Moreover, various SF sponge properties such as pore size, strength, and elasticity are controllable using our method.

Our earlier work has demonstrated that chondrocytes cultured on SF sponges show enhanced synthesis of extracellular matrices (ECMs) and glycosaminoglycan, which are the main components of cartilage tissue [11,12]. The results show that more chondrocytes on SF adhered to the surface strongly, migrated, and aggregated than chondrocytes on glass or fibronectin surfaces [11,12]. These results indicate that SF sponges support tissue regeneration as a useful cell scaffold. Furthermore, higher cell mobilities and gene expressions of ECM-related proteins were observed for fibroblasts cultured on SF films and sponges than for fibroblasts cultured on collagen and fibronectin [13]. Another study for which DNA microarray analysis was performed revealed higher gene expression in keratinocytes for skin reconstruction and wound healing on SF-based biomaterials than on collagen-based biomaterials [14], indicating that SF-based biomaterials have high potential for cell migration enhancement, for ECM production, and for wound repair. Cell migration at the wound site is known to improve rates of wound healing and implant therapy [15,16,17]. Moreover, cell migration is known to be related to various growth factors and cytokines during epithelialization in skin. Furthermore, regarding ECM production, a necessity for wound repair, SF-based biomaterials can support active cell migration to cells and can induce high ECM-related gene expression and differentiation at a contact site. Unlike collagen and fibronectin, SF has no bioactive sequence in protein chains such as Arg-Gly-Asp. Therefore, the unique material properties of SF-based biomaterials are expected to enhance such cell behaviors and to make them useful wound dressings.

This study evaluated the influence of SF-based biomaterials on cell migration and evaluated the gene expression of ECM and wound repair-related proteins in fibroblasts as human dermis model cells. After the SF films and sponges were prepared from aqueous solution, fibroblasts were cultured on these SF-based biomaterials. For the films, cell migration assays were performed using time-lapse imaging. After gene expression on the films was analyzed quantitatively using real-time reverse transcription polymerase chain reaction (RT-PCR), RT-PCR analysis was applied to fibroblasts cultured on three-dimensional sponges using commercially available collagen-based sponges as a control.

## 2. Results and Discussion

### 2.1. Cell Behaviors on SF Film

#### 2.1.1. Cell Migration and Morphology

**Figure 1:**
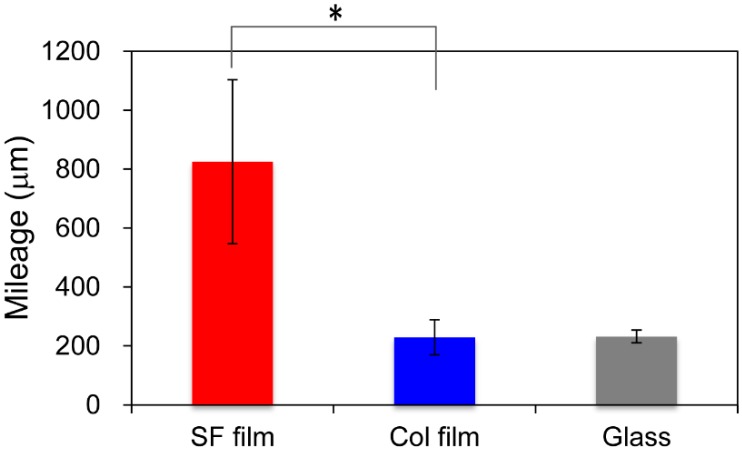
Quantitative cell mobility of fibroblasts. Using time-lapse microscopy, migration of fibroblasts on the silk fibroin (SF film), the collagen film (Col film), and glass surfaces was measured for 24 h at 5 min intervals. Pathways of fibroblasts were tracked using MtrackJ software for quantitative evaluation. * *p* < 0.05 Mileage indicates the total migration distance per cell for 24 h.

Several cells in time-lapse images were selected randomly and were traced using the MtrackJ system of the ImageJ software plug-in. Figure 1 shows migration distances for fibroblasts calculated for 24 h on three surfaces. Fibroblasts on the SF film migrated more actively than on collagen film (Col film) or glass surfaces. The average values of mileage were, respectively, 825.0, 229.3, and 232.0 μm. The migration rate for fibroblasts on the SF film was more than triple the value found for Col film and glass surfaces.

**Figure 2:**
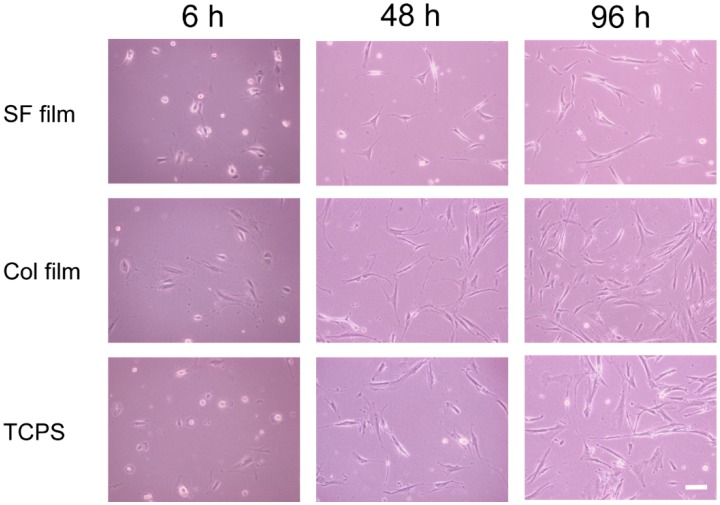
Morphologies of fibroblasts cultured on the SF film, Col film, and tissue culture polystyrene (TCPS) surfaces. Scale bar = 100 μm.

Figure 2 presents morphologies of attached fibroblasts at 6, 48, and 96 h after incubation on the SF film, Col film, and tissue culture polystyrene (TCPS) surfaces. After 6 h incubation on the SF film, relatively many fibroblasts adhered on the surface with round or spindle shapes. By contrast, fibroblasts extended to surfaces on the Col film. At 96 h incubation, almost all cells on the Col film and TCPS showed good spreading, which reflects strong cell adhesion, but cells on the SF film were observed to have various shapes, such as round and spindle-shaped. Round-shaped cells on the SF films were considered to be in the state of migration.

These results indicate that the surface properties of the SF film to support cell behaviors differ greatly from those of the Col film and TCPS. Interactions between cells and the SF film surface might be weaker than those on the Col film and TCPS surfaces. Collagen has a cell adhesive sequence in the molecules: RGDS (Arg-Gly-Asp-Ser). Therefore, fibroblasts can adhere to collagen through strong interaction between the RGDS sequence and integrins on the cell membrane. Morphologies of fibroblasts on the Col film were similar to those described in earlier reports of the relevant literature [18,19]. However, because SF film and TCPS have no such cell adhesive sequences, no bioactive interaction among them is expected. Nevertheless, cell migration and shapes on the SF film differed drastically from those on the TCPS, a typical cell culture substrate which is pre-treated with plasma irradiation for promoting the adhesions of cells. This finding indicates that the SF surface has characteristic properties that support the high cell migration behavior.

#### 2.1.2. Gene Expression on Films

**Figure 3:**
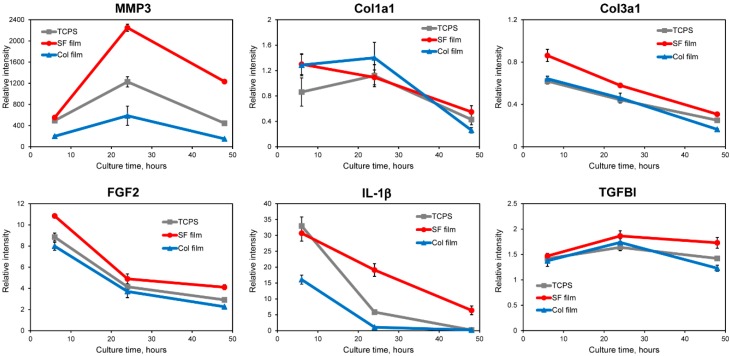
Relative mRNA expression levels of MMP3, Col1a1, Col3a1, FGF2, IL-1β, and TGFBI in fibroblasts cultured on the SF film, Col film, and TCPS after 6, 24, and 48 h culture. Quantitative RT-PCR was performed on total RNA from fibroblasts. All dCt values were corrected for the efficiency of the respective primer sets in relation to the GAPDH housekeeping gene.

To elucidate gene expression profiles of wound repair-related proteins in cultured fibroblasts at the initial stage of contact on the SF film, Col film, and TCPS surfaces, quantitative RT-PCR experiments were conducted. Figure 3 depicts the relative gene expression levels of various proteins in fibroblasts.

Fibroblasts produce matrix metallopeptidases (MMPs) to degrade ECMs during wound healing [20]. First, gene expressions of the protease and the ECM component were investigated. Matrix metallopeptidase 3 (MMP3) is reportedly involved in wound healing and tissue remodeling. It is necessary for initiation of the tissue reconstruction response [21], showing delayed wound healing observed in MMP3-knockout mice. In Figure 3, MMP3 expressions were up-regulated in fibroblasts cultured on all surfaces. Especially, the most up-regulated expression was observed on the SF film surface after 24 h culture. Collagen, type III, alpha 1 (Col3a1) and Collagen, type I, alpha 1 (Col1a1) are known to be major ECM components of skin dermal tissues. The gene expression of Col3a1 on the SF film surface was higher than on the Col film or TCPS surfaces. The Col3a1 gene was down-regulated on all surfaces throughout the cell culture. No significant difference between Col1a1 gene expressions was found on the three surfaces. The expression was kept at the same level until 24 h, after which it was decreased at 48 h culture. Karrer et al. reported that photodynamic therapy induced enhanced gene expression of MMP3 in fibroblasts, whereas the Col1a1 mRNA level was decreased at different time points (12–72 h); they concluded that induction of MMPs together with a reduction in collagen production might be useful to increase anti-sclerotic effects [22,23]. Therefore, we infer that the SF film induces acceleration of normal dermal tissue reconstruction, thereby diminishing scar formation. 

Next, gene expression profiles of wound-related growth factor and cytokine were evaluated. Fibroblast growth factor 2 (FGF2) is a mitogenic factor that is known to be expressed at the early stage at wound sites for healing. Gene expression of FGF2 was up-regulated. It decreased gradually on all surfaces over the entire time course. Compared to the Col film and TCPS surfaces, the SF film surface induced higher expression levels of FGF2. Interleukin 1, beta (IL-1β gene expressions were up-regulated on all surfaces compared with control after 6 h culture. Fibroblasts on the SF film and TCPS showed higher expression levels than those of cells on the Col film. After 24 h culture, the gene expression of IL-1β decreased gradually with increased culture time. At 48 h, the gene expression in fibroblasts on the Col film and TCPS surfaces returned to near control levels, whereas the SF film surface maintained up-regulated mRNA expression. IL-1β, which regulates the connective tissue metabolism, is related to the gene expression of collagens. Furthermore, results elucidating the IL-1β expression tendency similarly to our results were reported by Mercado et al.: gene expression of IL-1β was induced strongly after wounding. Moreover, expression levels decreased gradually and returned to near control levels at day 5 after wounding [24]. In the case of transforming growth factor, beta-induced (TGFBI) up-regulated gene expression was observed on all surfaces during 48 h culture. Relative gene expressions on the SF film were increased after 24 h and were maintained at the same level at 48 h. On the Col film and TCPS, TGFBI gene expressions were increased slightly before 24 h culture, but were decreased after 24 h. One ECM protein, TGFBI, is highly induced by TGF-β, which is known to be able to stimulate ECM synthesis and to increase fibroblast mobility with subsequent acceleration of wound healing and tissue construction [25,26,27]. In light of those reported findings, fibroblasts cultured on the SF film are expected to be activated during accelerated production of ECM proteins through TGFBI secretion during the early culture period. On the Col film, in contrast, ECM protein production is not facilitated in fibroblasts because the ECM environments were present at the initial culture period. Therefore, the Col film surface induced lower gene expression than the SF film surface did. Our earlier reports described studies using mouse fibroblasts for which cells expressed a higher TGFBI gene on the SF film than those of fibroblasts on the Col film or fibronectin film [13].

### 2.2. Cell Behaviors on SF Sponge

#### 2.2.1. Sponge Morphology and Properties

**Figure 4:**
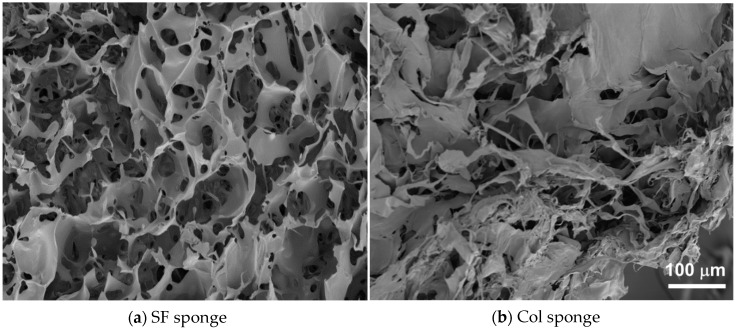
SEM images of SF sponge (**a**) and Col sponge (**b**).

Using our original method, we prepared three-dimensional porous SF sponges [9]. The SEM images reported in Figure 4a,b respectively depict the morphologies of cross-sections of an SF sponge and a commercially available collagen sponge (Col sponge). The SEM images revealed that both the SF sponge and Col sponge showed highly porous and interconnected structures. Actually, the SF sponge had an open and porous structure with 58 ± 19.0 μm pore size and homogeneous distribution of the pores throughout the sponge (Figure 4a), whereas the commercially available Col sponge showed a more irregular pore size distribution (Figure 4b). Mechanical properties of SF sponges under a wet state were investigated by measuring the compression strength. The Young′s modulus of the SF sponge was measured as 16 ± 1.2 kPa under wet conditions. Pailler-Mattei et al. evaluated the Young′s modulus of human skin as estimated between 4.5 and 8 kPa [28]. Actually, the SF sponge is slightly harder than living skin. However, as a wound dressing, the material must adhere tightly to cover the wound site and deform along with skin movements. Therefore, the SF sponge property is expected to be beneficial for use as a wound dressing material. The compressive strength of the Col sponge under a wet state was difficult to measure because of gelation in the presence of water. This property presents shortcomings as a wound dressing material because it is difficult to maintain the shape at the wound site and because it is difficult to handle during treatment. Typical collagen-based sponge wound dressing requires support materials such as silicone sheeting to ease its handling.

#### 2.2.2. Gene Expression on Sponge

**Figure 5:**
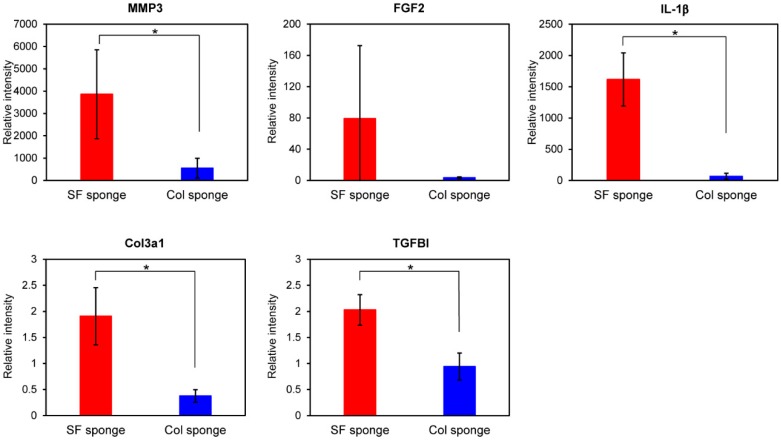
Gene expression levels of MMP, FGF2, IL-1β, Col3a1, and TGFBI in WI-38 fibroblasts cultured in the SF sponge and Col sponge after 48 h culture. Quantitative RT-PCR was performed for total RNA from fibroblasts. All dCt values were corrected for the efficiency of each primer set in relation to the housekeeping gene, GAPDH (* *p* < 0.05).

For gene expression analysis in fibroblasts cultured on sponges, we selected several genes as target genes. They are important for wound repair and skin reconstruction when the skin is wounded. Figure 5 presents the relative gene expressions of MMP3, FGF2, IL-1β Col3a1, and TGFBI at 48 h culture. Higher gene expressions of all genes we examined were observed in fibroblast culture on the SF sponge than on the Col sponge. Figure 5 shows that the expression level of MMP3 gene was found to be much higher than the other genes, similarly to the case of film substrates. Because, as described in 2.1.2, MMP3 is strongly related to normal tissue reconstruction, this result indicates that both sponges can strongly support tissue reconstruction with fibroblasts as a scaffold. However, the SF sponge is expected to induce the repair of tissues more effectively than the Col sponge. Furthermore, gene expressions of FGF2 and IL-1β were up-regulated in both sponges at 48 h culture. Gene expression levels of both cytokines in the SF sponge were 22-fold to 24-fold higher than in the Col sponge. Moreover, the relative gene expressions of Col3a1 and TGFBI were up-regulated considerably in SF the sponge, although down-regulation (Col3a1) and no change (TGFBI) of gene expressions were observed for the Col sponge. Up-regulation of these genes indicates induction and production of ECM proteins. Actually, ECM production is a major event in fibroblasts during wound healing. As shown in Figure 3, higher gene expression was observed on the SF film than on the Col film, as confirmed for the SF sponge. These results indicate that fibroblasts are induced to accelerate wound repair and skin reconstruction on and in SF-based biomaterials more effectively than on collagen-based biomaterials. Wound dressings must protect and maintain suitably moist environments at the wound site to achieve effective healing throughout normal repair processes. Quantitative analyses of migrations and ECM productions of mouse fibroblasts on the SF surface revealed that fibroblasts on the SF surface migrated more actively and showed higher gene expression of ECM-related proteins than these on the collagen or fibronectin surfaces [12]. In the case of human keratinocytes, cells migrated more actively on the SF surfaces than on the Col surface. The results of qRT-PCR and DNA microarray analyses showed that our SF sponges demonstrated the possibility of accelerated skin epithelialization, reconstruction, and wound repair with less formation of scar tissue during the wound healing processes [13]. Silk fibroin sponges can be anticipated for use as a wound dressing for effective wound repair and tissue reconstruction.

## 3. Materials and Methods

### 3.1. Preparation of Films and Sponges

*Bombyx mori* silkworm cocoons, which were kindly donated by Dr. Chiyuki Takabayashi (National Institute of Agrobiological Sciences, Okaya, Nagano, Japan), were degummed in 20 mM Na_2_CO_3_ at 95 °C for 30 min to remove the sericin layer. Silk fibroin (SF) aqueous solution was prepared from degummed silk fiber. For the SF film, SF aqueous solutions (1 *w*/*v* %) were poured into TCPS/glass-bottom dishes (AGC Techno Glass, Shizuoka, Japan). They were then incubated for 30 min at 25 °C. Subsequently, the solutions were removed. Then, the dishes were incubated in a dry oven at 50 °C for at least 12 h, with subsequent treatment with 80% MeOH solution for 30 min at 25 °C, for insolubilization of water-soluble SF films by crystallization. Finally, the dishes were dried at 50 °C for at least 12 h. Silk fibroin sponges were prepared using our freeze–thaw method as reported earlier [9,13]. Briefly, 1% *v*/*v* of DMSO, a sponge formation reagent, was mixed gradually to 4% *w*/*v* of SF aqueous solution. The mixed solution was poured into the aluminum mold (50 × 50 × 2 mm). The mold was immersed in 70% ethanol as a brine, where it was cooled gradually to −20 °C in a thermoregulated bath (EYELA, Tokyo, Japan). Then, it was kept for 17 h. The mold was thawed at 25 °C. After the obtained sponge was washed with pure water to remove DMSO, it was stored in water at 4 °C until further measurements. Before cell culture experiments, the SF films were disinfected using 70% EtOH. Then, the SF sponges were cut round to 6 mm diameter using a biopsy punch (Kai Corp., Tokyo, Japan). It was immersed in distilled water and was then autoclaved at 121 °C for 20 min.

Collagen solutions (0.3 mg/mL) were prepared by dilution of Cellmatrix Type I-C (Nitta Gelatin Inc., Osaka, Japan) with 0.22 μm filtered 1 mM HCl. They were then applied to TCPS/glass-bottom dishes at 25 °C. After 30 min, solutions were removed. The Col films were washed three times with sterilized PBS. All operations of the Col film preparations were done in sterile conditions. Collagen substrates covered with a silicon sheet (Terudermis; Olympus Terumo Biomaterials Corp., Tokyo, Japan) were kindly donated by Prof. Hajime Inoue (St. Marianna University School of Medicine, Kawasaki, Kanagawa, Japan) and were used as Col sponges.

The SEM images of the SF sponge and Col sponge were obtained using a scanning electron microscope (6301F; JEOL, Tokyo, Japan) with accelerating voltage of 5.0 kV. The average pore size was calculated by measuring 50 pores using ImageJ software (NIH, Bethesda, MD, USA).

### 3.2. Mechanical Test

The compressive modulus of the SF sponges (thickness: 5 mm) was measured using an EZ test (Shimadzu Corp., Kyoto, Japan) with a 10 N load cell. We compressed the SF sponges after the absorbing into water using an 8 mmφ disk apparatus at 5 mm/min at 25 °C (n = 8). The compressive modulus was calculated from the initial slope in the stress–strain curve.

### 3.3. Cell Culture and Migration

Cell migration on films was performed using the human WI-38 fibroblast cell line (Riken BRC, Tsukuba, Japan). The WI-38 were grown in Eagle’s medium (EMEM; Nissui, Japan) containing 10% FBS (Invitrogen Life Technologies Japan Ltd., Tokyo, Japan), 2 mM L-glutamine (Invitrogen Life Technologies Corp., USA), and 0.1 mg/mL kanamycin (Invitrogen Life Technologies Corp., USA) in a humidified incubator (5% CO_2_ and 95% air at 37 °C).

Then, the WI-38 fibroblasts were seeded on the SF film, Col film, and the glass bottom dish at densities of 7.5 × 10^4^ cells/dish. After 1 h incubation, unattached cells were removed. Then, films were placed in a chamber for microscopy (Olympus IX 70; Olympus Corp., Tokyo, Japan) with controlled temperature (37 °C) and CO_2_ gas concentration (5%). Time-lapse microscopic images were taken using a high sensitivity cool CCD color camera (Keyence Co., Tokyo, Japan) for 24 h at 5 min intervals. Migration pathways of WI-38 fibroblasts on surfaces were tracked using MtrackJ software (http://www.imagescience.org/meijering/software/mtrackj/) for quantitative evaluation [12,13]. Briefly, images with 289 slices were opened in ImageJ software. Then, MtrackJ plug-in software was used for manual tracking. Average cell speeds and mileages were calculated using data from five individual cells.

### 3.4. Quantitative RT-PCR

We seeded WI-38 fibroblasts on the SF film, Col film, and TCPS surfaces at densities of 5.0 × 10^4^ cells per dish. For sponges, fibroblasts were seeded on SF sponge and Col sponge at densities of 1.0 × 10^5^ cells per sponge. Cell cultures were grown in growth medium at 37 °C and 5% CO_2_. The medium was exchanged every 2 days. After 6, 24, 48, and 168 h culture, total RNA was recovered from cells using a kit (TaqMan^®^ Gene Expression Cells-to-CT^TM^; Ambion Life Technologies Japan Ltd., Tokyo, Japan). PCR experiments were conducted using StepOne (Applied Biosystems; Life Technologies Japan Ltd., Tokyo, Japan). Data were normalized against the CT of the human glyceraldehyde-3-phosphate dehydrogenase (GAPDH) housekeeping gene. Relative mRNA expressions were analyzed using the ddCT equation. As target genes, matrix metallopeptidase 3 (MMP3, Hs00968305_m1), collagen, type III, alpha 1 (Col31, Hs00943809_m1), fibroblast growth factor 2 (FGF2, Hs00266645_m1), interleukin 1, beta (IL-1β, Hs01555410_m1), collagen, type I, alpha 1 (Cola1, Hs01076777_m1) and transforming growth factor, beta-induced (TGFBI, Hs00932747_m1) were selected.

## 4. Conclusions

This study compared WI-38 fibroblast behaviors on SF-based biomaterial with those on collagen-based biomaterial. Higher cell migration on the SF film than on the Col film was observed. Cells cultured on the SF film exhibited higher gene expression related to wound repair and skin reconstruction than on the Col film. Moreover, ECM-production-related genes were up-regulated in WI-38 fibroblasts cultured in the SF sponge, and higher expression of the genes was observed than on the Col sponge. These results indicate the SF materials as a useful biomaterial for wound dressing: the SF materials hasten tissue reconstruction.
